# Effect of Point Spread Function Deconvolution in Reconstruction of Brain ^18^F-FDG PET Images on the Diagnostic Thinking Efficacy in Alzheimer's Disease

**DOI:** 10.3389/fmed.2021.721551

**Published:** 2021-07-29

**Authors:** Matthieu Doyen, Elise Mairal, Manon Bordonne, Timothée Zaragori, Véronique Roch, Laetitia Imbert, Antoine Verger

**Affiliations:** ^1^Université de Lorraine, IADI, INSERM U1254 and Nancyclotep Imaging Platform, Nancy, France; ^2^Department of Nuclear Medicine, Université de Lorraine, CHRU Nancy, Nancy, France

**Keywords:** point spread function, FDG PET, Alzheimer's disease, digital, diagnostic thinking efficacy

## Abstract

**Purpose:** This study aims to determine the effect of applying Point Spread Function (PSF) deconvolution, which is known to improve contrast and spatial resolution in brain ^18^F-FDG PET images, to the diagnostic thinking efficacy in Alzheimer's disease (AD).

**Methods:** We compared Hoffman 3-D brain phantom images reconstructed with or without PSF. The effect of PSF deconvolution on AD diagnostic clinical performance was determined from digital brain ^18^F-FDG PET images of AD (*n* = 38) and healthy (*n* = 35) subjects compared to controls (*n* = 36). Performances were assessed with SPM at the group level (*p* < 0.001 for the voxel) and at the individual level by visual interpretation of SPM T-maps (*p* < 0.005 for the voxel) by the consensual analysis of three experienced raters.

**Results:** A mix of large hypometabolic (1,483cm^3^, mean value of −867 ± 492 Bq/ml) and intense hypermetabolic (902 cm^3^, mean value of 1,623 ± 1,242 Bq/ml) areas was observed in the PSF compared to the no PSF phantom images. Significant hypometabolic areas were observed in the AD group compared to the controls, for reconstructions with and without PSF (respectively 23.7 and 26.2 cm^3^), whereas no significant hypometabolic areas were observed when comparing the group of healthy subjects to the control group. At the individual level, no significant differences in diagnostic performances for discriminating AD were observed visually (sensitivity of 89 and 92% for reconstructions with and without PSF respectively, similar specificity of 74%).

**Conclusion:** Diagnostic thinking efficacy performances for diagnosing AD are similar for ^18^F-FDG PET images reconstructed with or without PSF.

## Introduction

Recently, significant improvements have been performed in the iterative reconstruction algorithms to enhance the image quality but also the accuracy of the quantification. Particularly, these methods include a resolution modeling or point-spread function (PSF) available on all vendors of clinical PET/CT systems. This PSF allows to correct from the system's depiction of point sources depending on their location in the field of view (1).

Application of this novel PSF reconstruction algorithm to brain ^18^F-FDG PET imaging improves image quality by enhancing contrast and spatial resolution (2). However, PSF deconvolution increases image noise and can lead to an overestimation of the real activity concentration, namely edge or Gibbs' artifacts, thereby limiting its use in routine practice.

Visual evaluation as well as semi-quantitative analyses of brain ^18^F-FDG PET imaging are compelling for the diagnosis of neurodegenerative disorders such as Alzheimer's disease (AD) (3). Indeed, a hypometabolic pattern involving the posterior temporo-parietal association cortex is typically reported in AD (3). Reconstructions with PSF improve lesion detectability and are generally associated with an increase in sensitivity while slightly reducing specificity. To the best of our knowledge, no study has to date investigated the application of PSF reconstructions on brain ^18^F-FDG PET images in the diagnostic thinking efficacy in AD. Reconstructions with PSF have predominantly been studied in the context of focal lesions (4). Although we know that AD is characterized by diffuse hypometabolism (3), the effects of reconstructions with PSF in AD are poorly understood. In addition, the lack of harmonization of reconstruction protocols for brain PET imaging currently limits multi-center collaborations (4).

Our study therefore aims to elucidate the clinical impact of PSF deconvolution, applied to brain ^18^F-FDG PET imaging, in the diagnosis of AD.

## Materials and Methods

### Phantom

A 15 min-acquisition of a Hoffman 3-D brain phantom, filled with 76.4 MBq of ^18^F, was performed on a digital camera (Vereos, Philips^®^) and then reconstructed with or without the PSF deconvolution. Spatially PET normalized images of the Montreal National Institute (MNI) space with and without PSF deconvolution were compared by subtraction, after inclusive whole-brain masking. PSF deconvolution parameters (1 iteration and 6-mm regularization kernel) were optimized according to the noise level and the gray/white matter contrast ratio (2) (Supplementary Figure 1).

### Patients

We selected three groups of subjects that had undergone brain ^18^F-FDG PET on a digital camera (Vereos, Philips^®^) with a similar acquisition protocol and reported reconstruction protocol derived from the guidelines (5, 6). Briefly, brain ^18^F-FDG PET scans were recorded over a 15 min one bed acquisition, 45–50 min after injection of 2 MBq/kg of ^18^F-FDG. All subjects had fasted at least 6 h prior to receiving the injection and had blood glucose levels <160 mg/dl. All PET images were reconstructed with iterative OSEM methods, as performed in routine clinical practice, and corrected for scatter, random and attenuation with a CT scan. Reconstructed parameters included two iterations and 10 subsets, a Gaussian post- filter (4.0 mm FWHM) with a 256 mm reconstruction diameter and a 1-mm^3^ voxel size. The AD group was retrospectively constituted from a group of patients referred for evaluation of a cognitive complaint between March 2019 and November 2020 and who exhibited additional positive cerebro-spinal fluid biomarkers according to the NIA-AA-2018 classification (7). The other groups of heathy (H) and control subjects, were derived from the prospective NCT03345290 study, they did not have a neurological disease and were matched for age, sex, Mini Mental State Examination (MMSE) and educational level. All PET images were reconstructed with or without PSF deconvolution using the previously optimized parameters.

### Statistical Parametric Mapping

Brain ^18^F-FDG PET images were pre-processed using SPM12 (London, UK). All ^18^F-FDG PET images, reconstructed with or without the PSF deconvolution, were spatially normalized with an adaptive CT template using CT attenuation corrected images and then corrected for partial volume effects (5). The pons region was used as reference for intensity normalization (8). All analyses were performed with two-sample *t*-tests with an inclusive AD mask. AD and H groups were compared to the control group twice, once with and then without PSF deconvolution (search for decreased metabolism with *p* < 0.001 for the voxel, cluster volume corrected for the expected volume provided by SPM with age and sex as covariates). Each subject from the AD and H group was individually compared to the control group (search for decreased metabolism with *p* < 0.005 for the voxel, cluster volume corrected for the expected volume provided by SPM) this was done for both images with and without the PSF deconvolution. Three experienced observers (MB, EM, and AV), who were blinded to the patients' clinical data, gave a dichotomous reading: AD diagnosis or not, after reviewing the SPM T-maps. A pattern of diffuse hypometabolism within the regions known to be involved in AD was considered a positive scan (3). Results were expressed as a consensual analysis for the positive diagnosis of AD.

### Statistical Analysis

Categorical variables are expressed as percentages and continuous variables as means and standard deviations. Due to the non-normality of variable distributions, Chi-2 and Kruskal–Wallis tests were performed for comparisons of categorical and continuous variables, respectively. For the comparisons of diagnostic performances at the individual level, Mc Nemar tests were used with corrections for multiple comparisons. A *p*-value < 0.05 was considered to be significant. All tests were performed with SPSS (SPSS Statistics for Windows, Version 20.0. Armonk, NY: IBM Corp).

## Results

### Brain Phantom

**Figure 1:**
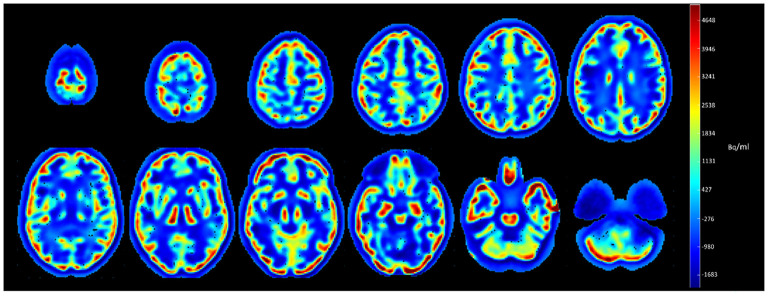
Subtracted axial slices of Hoffman 3-D brain phantom images reconstructed with and without PSF.

A mix, of large hypometabolic (1,483 cm^3^, mean value of −867 ± 492 Bq/ml) and intense hypermetabolic (903 cm^3^, mean value of 1,623 ± 1,243 Bq/ml) areas, was observed in PSF images compared to the no PSF images (Figure 1).

### Population

The study included 109 subjects. As detailed in Table 1, no differences in age, sex, MMSE, educational level and blood glucose level were observed between healthy subjects and controls. However, AD patients were significantly older and exhibited lower levels of education, MMSE compared to the other two groups (*p* ≤ 0.03). Moreover, even if all subjects included in this study presented normal levels of blood glucose (<160 mg/dL), higher levels were observed in AD patients as compared to controls (*p* = 0.02).

**Table 1:** Characteristics of Alzheimer's disease (AD), healthy (H) group patients and controls.

**Group**	**AD (*n* = 38)**	**H (*n* = 35)**	**Controls (*n* = 36)**	***p*-values**
Age (years old)	71.6 ± 7.4^¥^	55.5 ± 17.5	55.9 ± 16.7	<0.01^*^
Sex (Female)	17 (44 %)	19 (54 %)	18 (50 %)	0.72
Educational level				0.03^*^
None	0	0	0	
Primary School	11^¥^	4	7	
High School	18^¥^	12	9	
College	9^¥^	19	19	
MMSE	17.4 ± 6.3^¥^	29.0 ± 1.0	28.7 ± 0.9	<0.01^*^
Blood glucose level (mg/dL)	101.9 ± 14.4^§^	100 ± 21.7	92 ± 14.2	0.02^*^

*MMSE, Mini-mental state examination;*

* *p-value significant for the comparison between the three groups;*

¥ *group significantly different from the two others;*

§ *group significantly different from the controls*.

### Image Comparisons at the Group Level

**Figure 2:**
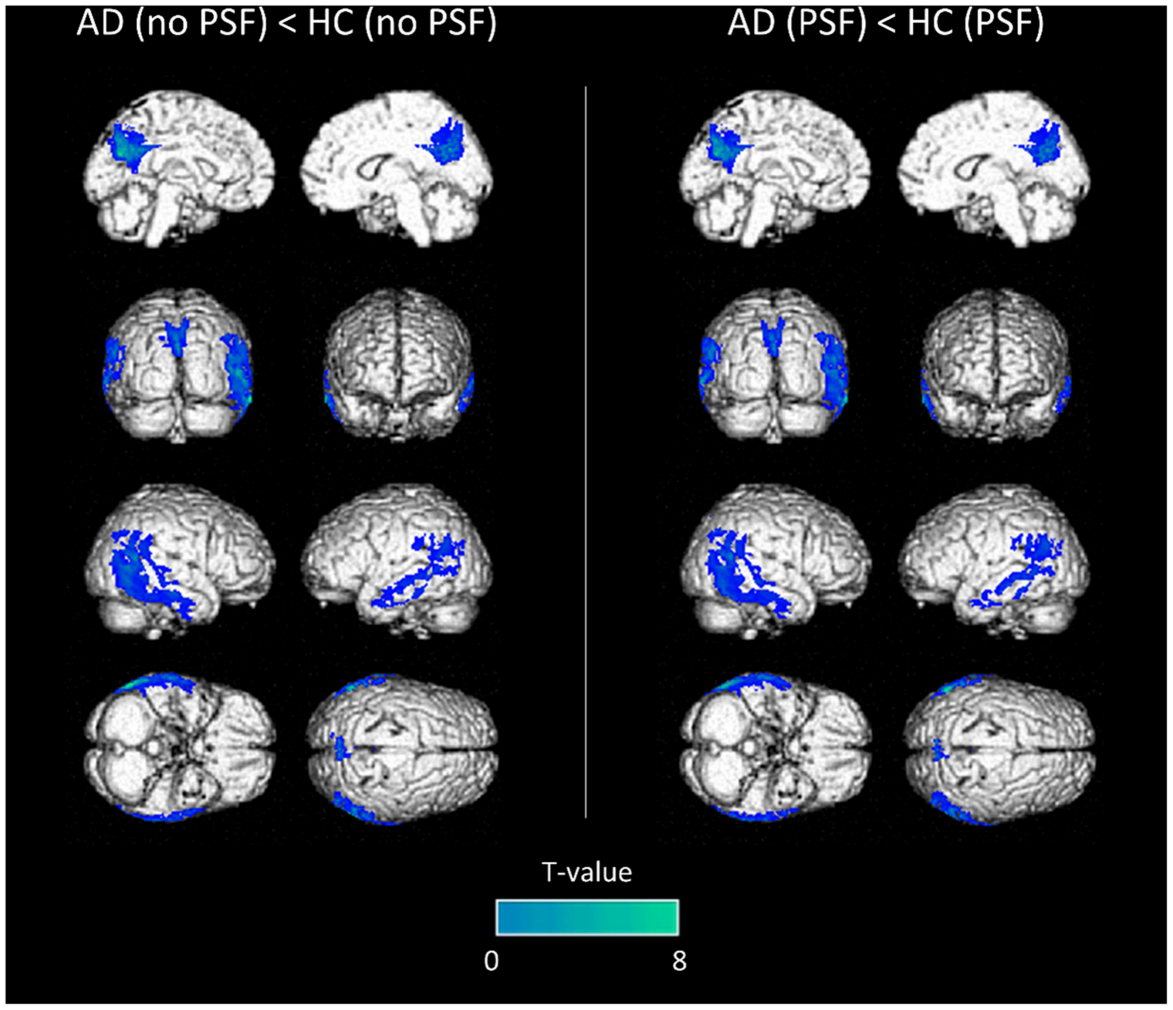
Anatomical localization of areas of decreased metabolic activity in AD patients compared to controls (*p* < 0.001, uncorrected, *k* > 1.5 cm^3^) from PET images without PSF deconvolution (left panel) or with (right panel), projected onto 3D volume rendering, spatially normalized and smoothed into the Montreal National Institute (MNI) space.

Significant hypometabolic regions were observed in AD subjects when compared to the controls with or without PSF (23.7 cm^3^ with a T-voxel max value of significance of 7.09, vs. 26.2 cm^3^ with a T-voxel max value of significance of 8.63 respectively, Figure 2). No significant hypometabolic areas were observed for either types of reconstructions when healthy subjects were compared to the controls.

### Image Comparisons at the Individual Level

Similar performances were observed in the visual analysis when using the two datasets of images for the diagnosis of AD with respective sensitivity, specificity and accuracy of 92.1, 74.3, 83.6%, for images reconstructed without PSF, and 89.5, 74.3, 82.2%, for images reconstructed with PSF (*p* = 1.0). Only one case was discordant between the two reconstructions (one false negative AD case identified with the PSF reconstruction).

**Figure 3:**
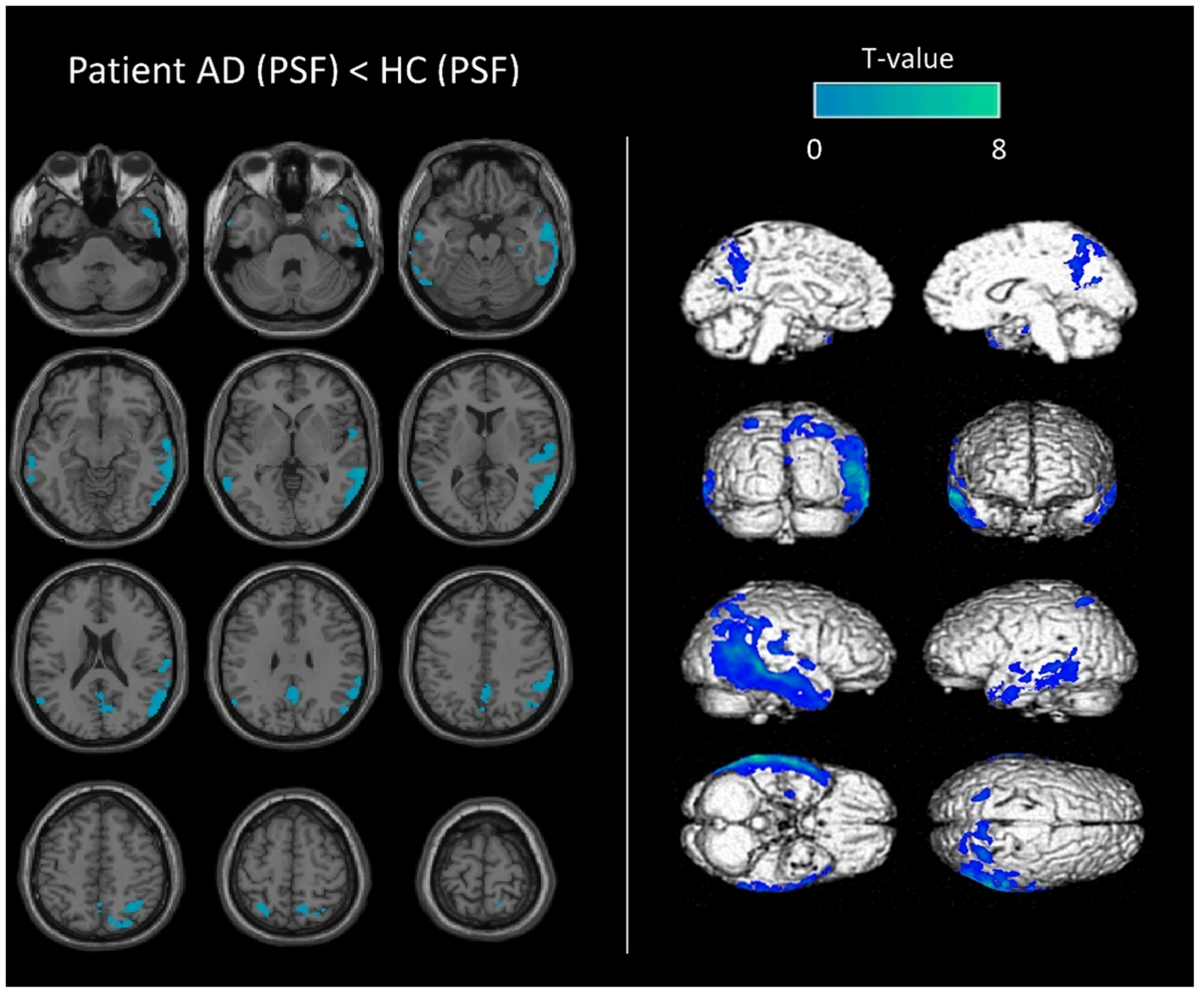
Representative SPM-T map images used in the visual analysis. Anatomical localization of decreased metabolic activity in an AD patient (79-year-old man with MMSE score of 10), compared to controls. SPM-T maps are projected onto two-dimensional slices of T1-weighted MRIs (from the base to the top of the skull, left panel) and 3D-rendered volumes (right panel). Images used in this example (AD patient and controls group) were reconstructed using PSF deconvolution.

Representative SPMT-maps used for visual analysis are shown in Figure 3.

## Discussion

The present study shows that brain ^18^F-FDG PET images reconstructed with or without PSF yield similar overall AD diagnostic thinking performances.

Our preliminary results on the phantom showed that the effects of reconstructions with PSF were not homogeneous across the whole brain volume, and as expected with increased gray-to-white matter contrast ratios (4). Importantly, our voxel-to-voxel wise approach after subtraction of the two normalized images highlighted that this contrast enhancement was mainly observed in the peripheral gray-matter regions, with maximum enhancement coinciding with the maximal contrast ratio, i.e., at the interface of the cerebral cortex and the extracerebral regions (Figure 1).

Our clinical analysis showed similar findings at the group level between paired PET images reconstructed with or without PSF. In addition, diagnostic thinking efficacy at the individual level showed overall similar performances between both reconstructions. This is in contrast to a previous study (9) which reported significant differences in absolute *z*-scores obtained with an automated software when applying PSF reconstructions in neurodegenerative diseases. However, this particular study did not perform reconstructions on the normal database which was provided by the software. This is in contrast to our own study which exclusively compared populations with the same PSF status, given that our normal database was reconstructed with or without PSF depending on the nature of the population investigated (5). It has been previously suggested that PET images should be smoothed to the same low spatial resolution to assess comparability between images (10). However, our current study, only applies a Gaussian post-reconstruction filter adapted to the spatial resolution of a digital PET. Moreover, our AD patients, were selected on cerebro-spinal fluid biomarkers and were representative of patients encountered in daily clinical practice, exhibiting different stages of typical and atypical Alzheimer's disease. This could in part explain why a false negative AD case was misdiagnosed with the PSF reconstruction, a “non-perfect” 90% of detection sensitivity being typically reported for brain ^18^F-FDG PET in AD (3).

PSF enhances image quality thereby increasing visual reader comfort. Indeed, improvements in image contrast and spatial resolution facilitate the delineation of cortical gyri (2). In our study, local overestimations associated with the application of PSF did not influence the AD diagnosis, the diagnosis being related to the relative difference between brain region metabolism. Integrating PSF in reconstructions across PET/CT systems has already been suggested (4). The present study validates the application of PSF to brain ^18^F-FDG PET imaging as a diagnostic thinking efficacy, a confirmation that was previously lacking in the context of AD (1).

A limitation of our work is the application of reconstructions with PSF to a single PET system, which limits the generalization of our results to other manufacturers.

In light of the similar diagnostic AD thinking performances with and without PSF and the improved visual comfort of reconstructions integrating PSF, PSF can be deployed in clinical routine and used in multicenter neurodegenerative studies for protocol harmonization. The benefits of reconstructions with PSF in other brain pathologies and with other radiotracers remains to be established.

## Data Availability Statement

The raw data supporting the conclusions of this article will be made available by the authors, without undue reservation.

## Ethics Statement

The studies involving human participants were reviewed and approved by Subjects gave their written informed consent to participate in the study in accordance with the Declaration of Helsinki. This study was approved on January 18, 2021 by the local ethics committee (NCT04718207, ID Number S201215_051). The patients/participants provided their written informed consent to participate in this study. Written informed consent was obtained from the individual(s) for the publication of any potentially identifiable images or data included in this article.

## Author Contributions

MD, EM, and AV contributed to the writing of the manuscript. LI and AV contributed to the revision of the manuscript. All authors contributed significantly to the analysis and interpretation of the data.

## Conflict of Interest

The authors declare that the research was conducted in the absence of any commercial or financial relationships that could be construed as a potential conflict of interest.

## Publisher's Note

All claims expressed in this article are solely those of the authors and do not necessarily represent those of their affiliated organizations, or those of the publisher, the editors and the reviewers. Any product that may be evaluated in this article, or claim that may be made by its manufacturer, is not guaranteed or endorsed by the publisher.

## Supplementary Material

Supplementary Figure 1 Gray/white matter contrast between peripheral gray-matter (occiput) and white matter (semi-oval center) structures, according to the noise level within the semi-oval area for different PSF parameters. Among all combinations evaluated, only acquisitions that could be visually interpreted by an experienced physician were considered. PSF parameters selected for this study are represented by the red arrow. IT, iteration number; R, regularization kernel expressed in mm.

## References

[1] Rogasch JMM Boellaard R Pike L Borchmann P Johnson P Wolf J. Moving the goalposts while scoring-the dilemma posed by new PET technologies. Eur J Nucl Med Mol Imaging. (2021) 48:2696–710. 10.1007/s00259-021-05403-2 33990846 PMC8263433

[2] Salvadori J Odille F Verger A Olivier P Karcher G Marie P-Y. Head-to-head comparison between digital and analog PET of human and phantom images when optimized for maximizing the signal-to-noise ratio from small lesions. EJNMMI Phys. (2020) 7:11. 10.1186/s40658-020-0281-8 32086646 PMC7035408

[3] Nobili F Festari C Altomare D Agosta F Orini S Van Laere K. Automated assessment of FDG-PET for differential diagnosis in patients with neurodegenerative disorders. Eur J Nucl Med Mol Imaging. (2018) 45:1557–66. 10.1007/s00259-018-4030-3 29721650

[4] Verwer EE Golla SSV Kaalep A Lubberink M van Velden FHP Bettinardi V. Harmonisation of PET/CT contrast recovery performance for brain studies. Eur J Nucl Med Mol Imaging. (2021) 48:2856–70. 10.1007/s00259-021-05201-w 33517517 PMC8263427

[5] Mairal E Doyen M Rivasseau-Jonveaux T Malaplate C Guedj E Verger A. Clinical impact of digital and conventional PET control databases for semi-quantitative analysis of brain 18F-FDG digital PET scans. EJNMMI Res. (2020) 10:144. 10.1186/s13550-020-00733-y 33258085 PMC7704892

[6] Varrone A Asenbaum S Vander Borght T Booij J Nobili F Någren K. EANM procedure guidelines for PET brain imaging using [18F]FDG, version 2. Eur J Nucl Med Mol Imaging. (2009) 36:2103–10. 10.1007/s00259-009-1264-0 19838705

[7] Jack CR Bennett DA Blennow K Carrillo MC Dunn B Haeberlein SB. NIA-AA Research Framework: toward a biological definition of Alzheimer's disease. Alzheimers Dement J Alzheimers Assoc. (2018) 14:535–62. 10.1016/j.jalz.2018.02.018 29653606 PMC5958625

[8] Verger A Doyen M Campion JY Guedj E. The pons as reference region for intensity normalization in semi-quantitative analysis of brain 18FDG PET: application to metabolic changes related to ageing in conventional and digital control databases. EJNMMI Res. (2021) 11:31. 10.1186/s13550-021-00771-0 33761019 PMC7990981

[9] Lindström E Oddstig J Danfors T Jögi J Hansson O Lubberink M. Image reconstruction methods affect software-aided assessment of pathologies of [18F]flutemetamol and [18F]FDG brain-PET examinations in patients with neurodegenerative diseases. NeuroImage Clin. (2020) 28:102386. 10.1016/j.nicl.2020.102386 32882645 PMC7476314

[10] Joshi A Koeppe RA Fessler JA. Reducing between scanner differences in multi-center PET studies. NeuroImage. (2009) 46:154–9. 10.1016/j.neuroimage.2009.01.057 19457369 PMC4308413

