# Supplementary figures and images for: Effect of Point Spread Function Deconvolution in Reconstruction of Brain 18F-FDG PET Images on the Diagnostic Thinking Efficacy in Alzheimer's Disease
Source: Front Med (Lausanne). 2021 Jul 29;8:721551. doi: 10.3389/fmed.2021.721551 (PMC8358179; doi:10.3389/fmed.2021.721551)

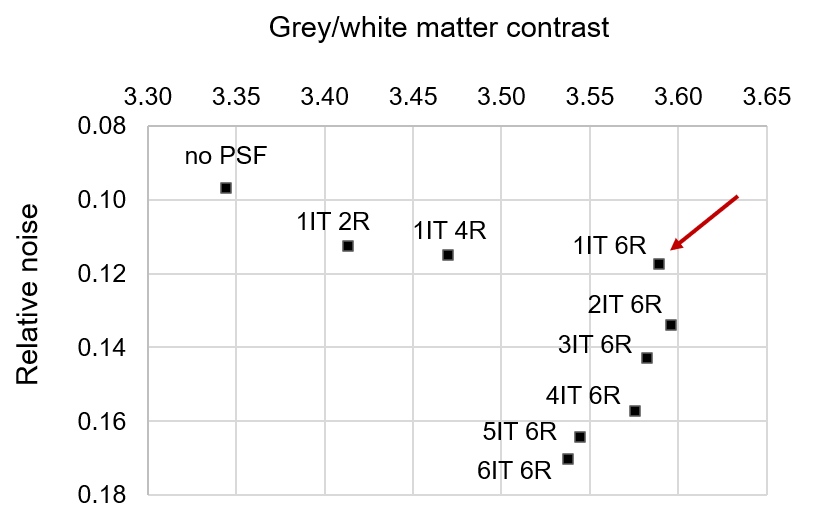

Supplement: Supplementary Figure 1 — Gray/white matter contrast between peripheral gray-matter (occiput) and white matter (semi-oval center) structures, according to the noise level within the semi-oval area for different PSF parameters. Among all combinations evaluated, only acquisitions that could be visually interpreted by an experienced physician were considered. PSF parameters selected for this study are represented by the red arrow. IT, iteration number; R, regularization kernel expressed in mm. [file Image_1.TIF]
